# A Mathematical Model for the Detection Mechanism of DNA Double-Strand Breaks Depending on Autophosphorylation of ATM

**DOI:** 10.1371/journal.pone.0005131

**Published:** 2009-04-13

**Authors:** Kazunari Mouri, Jose C. Nacher, Tatsuya Akutsu

**Affiliations:** 1 Bioinformatics Center, Institute for Chemical Research, Kyoto University, Gokasho Uji, Kyoto, Japan; 2 Department of Complex Systems, Future University-Hakodate, Kamedanakano-cho, Hakodate, Hokkaido, Japan; Brunel University, United Kingdom

## Abstract

**Background:**

After IR stress, DNA double-strand breaks (DSBs) occur and repair proteins (RPs) bind to them, generating DSB-RP complexes (DSBCs), which results in repaired DSBs (RDSBs). In recent experimental studies, it is suggested that the ATM proteins detect these DNA lesions depending on the autophosphorylation of ATM which exists as a dimer before phosphorylation. Interestingly, the ATM proteins can work as a sensor for a small number of DSBs (approximately 18 DSBs in a cell after exposure to IR). Thus the ATM proteins amplify the small input signals based on the phosphorylation of the ATM dimer proteins. The true DSB-detection mechanism depending on ATM autophosphorylation has yet to be clarified.

**Methodology/Principal Findings:**

We propose a mathematical model for the detection mechanism of DSBs by ATM. Our model includes both a DSB-repair mechanism and an ATM-phosphorylation mechanism. We model the former mechanism as a stochastic process, and obtain theoretical mean values of DSBs and DSBCs. In the latter mechanism, it is known that ATM autophosphorylates itself, and we find that the autophosphorylation induces bifurcation of the phosphorylated ATM (ATM*). The bifurcation diagram depends on the total concentration of ATM, which makes three types of steady state diagrams of ATM*: monostable, reversible bistable, and irreversible bistable. Bistability exists depending on the Hill coefficient in the equation of ATM autophosphorylation, and it emerges as the total concentration of ATM increases. Combining these two mechanisms, we find that ATM* exhibits switch-like behaviour in the presence of bistability, and the detection time after DNA damage decreases when the total concentration of ATM increases.

**Conclusions/Significance:**

This work provides a mathematical model that explains the DSB-detection mechanism depending on ATM autophosphorylation. These results indicate that positive auto-regulation works both as a sensor and amplifier of small input signals.

## Introduction

Recently, research suggests that biological functions depend on specific small components called network motifs [1]. In these motifs, positive and negative feedbacks are very important for bistability or oscillatory behaviours, respectively. For example, positive feedback in mitogen-activated protein kinase (MAPK) cascade produces bistability of phosphorylated MAPK which contributes to an all-or-none cell fate switch [2], [3], and the production of self-sustained biochemical “memories” of transient stimuli [4]. A synthetic regulatory network of a mutually inhibitory double negative feedback loop in *Escherichia coli* also provides bistability, and a simple theory that predicts the conditions necessary for bistability has been suggested [5]. Also, stochasticity of gene expression in a single cell has been recently observed [6]. These stochastic single-molecule events determine a cell's phenotype depending on positive feedback [7], [8], [9]. However, understandings of functions for these positive feedbacks are limited.

Generally, there are several factors which damage DNA in cells. Signal-transduction pathways are rapidly activated after exposure to DNA-damaging agents. *ATM*, the gene that is mutated in the human disease ataxia-telangiectasia (AT), is important for activating signalling pathways in mammalian cells following exposure to ionizing radiation (IR) or oxidative stress where DNA double strand breaks (DSBs) are generated [10]. For example, hydrogen peroxide (H_2_O_2_), one type of oxidative stress, is a normal metabolite in the cell whose steady-state concentration is in the range 10^−8^–10^−9^ M [11], [12], and is one of the products to protect the mammalian host from the invasion of bacillus [13]. However, if it is not properly controlled, it can cause severe damage to a cell. In the presence of Fe^2+^, H_2_O_2_ can generate free radical (OH^•^). Also, the Haber-Weiss reaction can form OH^•^ in an interaction between 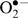 and H_2_O_2_ in the presence of Fe^2+^ or Fe^3+^ [12]. These oxygen free radicals and H_2_O_2_ are spoken of as reactive oxygen species (ROS). DNA strand breaks are due to free radicals in these reactions. Depending on its concentration, H_2_O_2_ induces two types of DNA lesions: DNA single- and double-strand breaks. DNA single-strand breaks (SSBs) are dominant under 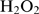 stress, but these lesions are efficiently repaired and do not appear to mediate the cytotoxic response [14]. On the other hand, DNA double-strand breaks (DSBs) seldom occur under 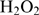 stress, but they are toxic and potentially induce apoptosis. In addition, after IR stress, DNA double-strand breaks are generated and ATM proteins are phosphorylated. The true mechanism of this process has not been understood yet. However, one of the targets of ATM phosphorylation is suggested to be the Nbs1 (nibrin) protein, which associates with the conserved DSB repair factors Mre11 and Rad50 [15]. The phosphorylated ATM phosphorylates itself, and it is suggested that ATM is autophosphorylated within 15 min after exposure to 0.5 Gy IR, which induces only 18 DNA breaks, approximately [16], [17]. However, it has not been known how ATM detects a small number of DSBs and activates signalling cascades. In this paper, we propose a mathematical model of the ATM phosphorylation process after a stochastic generation of a small number of DSBs regardless of the source of damage.

In our model, we assume that DSBs are generated under DNA damage, repair proteins (RPs) bind to them and become DSB-RP complexes (DSBCs), and DSBs are repaired. The numbers of DSBs and DSBCs are very small and these processes are stochastic. We will see that we can calculate theoretical values for the mean numbers of DSBs and DSBCs by using the number of repair proteins and rate constants of repair processes. The produced DSBs and DSBCs phosphorylate ATM which autophosphorylates itself. We will find that DSBs are not successfully repaired and the number of DSBs increases when the number of repair proteins is small, but when sufficient repair proteins exist, the number of DSBs is suppressed to low levels. Also, we will find that autophosphorylation of ATM induces bifurcation of the phosphorylated ATM (ATM*). Depending on the total concentration of ATM, the fixed points of ATM* will have three types of steady state diagrams: monostable, reversible bistable, and irreversible bistable diagrams. Of these steady-state diagrams, bistability emerges when the total concentration of ATM increases, and the concentration of ATM* exhibits switch-like behaviour in the presence of such bistabilities. Furthermore, we will see that the time to detection after the DNA damage decreases when the total concentration of ATM increases.

## Results

**Figure 1:**
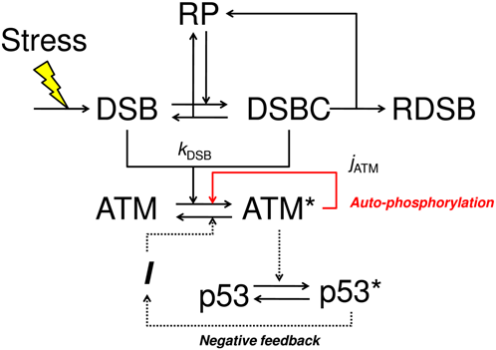
A diagram of the detection mechanism of DSB depending on ATM. Abbreviations – DSB: DNA double-strand break; RP: repair protein; DSBC: DNA double-strand break and repair protein complex; RDSB: repaired DSB. Asterisks denote phosphorylated proteins. *I* denotes an intermediate repressor of ATM. Some stress signal induces DSBs which are repaired by RPs. ATM detects DSBs and DSBCs, and then it is phosphorylated. The red arrow denotes autophosphorylation of ATM, which amplifies the stress signal. The dotted arrows denote a negative feedback loop. We mainly address DSB repair processes and the ATM sensor module. We refer to the effect of the negative feedback from p53 only in the Discussion section. Details are shown in the main text.

Figure 1 shows a diagram of our model. DSBs are induced by some stress signal, and repair proteins bind to DSBs, generating DSBCs. The repaired complex produces a repaired DSB. We model these processes as stochastic processes. Details are shown in the next section. The generated DSBs and DSBCs are detected by ATM, and it phosphorylates itself to amplify the stress signal. In this paper, we mainly focus on the repair processes of DSBs and the detection mechanism of ATM. The negative feedback from p53 is also treated in the Discussion section.

### Calculation methods for DSB-production model

**Figure 2:**
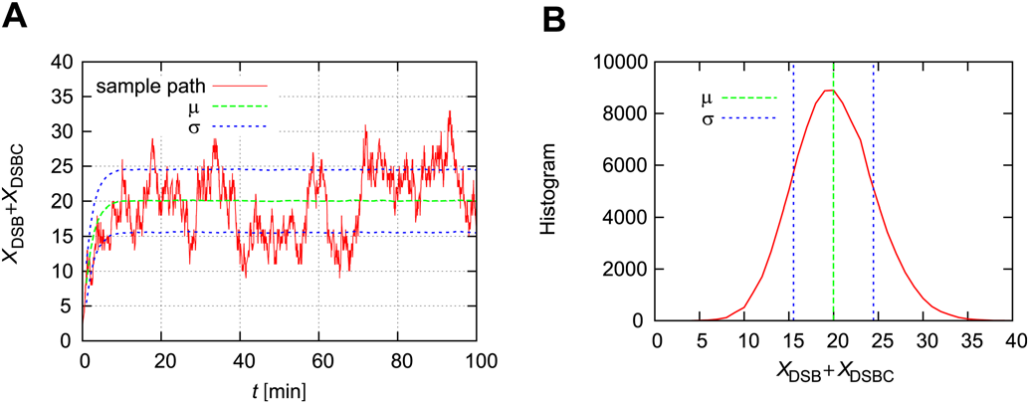
Simulation results of repair processes of DSBs. (A) A time course of the numbers of DSBs and DSBCs with the time-dependent expectation (μ) and standard deviation (σ). The blue lines denote μ±σ. (B) The histogram of the numbers of DSBs and DSBCs at time *t* = 100[min]. In these figures, the maximum number of repair proteins 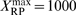. Other parameters are as defined in Table 1. Initial numbers of molecules are 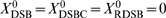, and 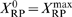.

In this paper, we assume the following schemes of a stochastic production mechanism of DSBs and their repair processes:
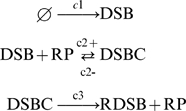
(1)where DSB denotes DNA double strand breaks, RP denotes repair proteins, DSBC denotes DSB and repair protein complexes, and RDSB denotes the repaired DSB. The constants, 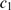, 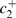, 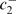, 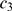, represent the stochastic rate constants [18] (or reaction parameters [19]). Also, the associated rate laws (or hazard functions) are 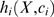, where *i* is a reaction type and 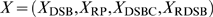 is the current state (the number of molecules (or sites) of each reaction species) of the system. These chemical reactions occur stochastically, thus the fluctuations of the number of molecules which are produced in these reactions are stochastic processes [18]. For example, the production of DSBs is a zeroth-order reaction, and the hazard of the reaction is
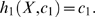
(2)The repair process of the DSBs is a second-order reaction, and the combined hazard of the reaction is
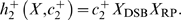
(3)The failed and succeeded repair processes are first-order reactions, and we respectively denote the combined hazards of each reaction as
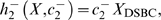
(4)

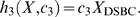
(5)The above equations allow us to calculate 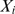 for each molecular type *i* by using the Gillespie algorithm (we use Gillespie's direct method [20]). For example, a time course of 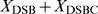 is shown in Figure 2. When there are a large number of repair proteins (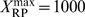), the numbers of DSBs and DSBCs are fluctuating with a mean μ∼20 and standard deviation σ∼4.5.

### Comparison of theoretical and simulation results

**Figure 3:**
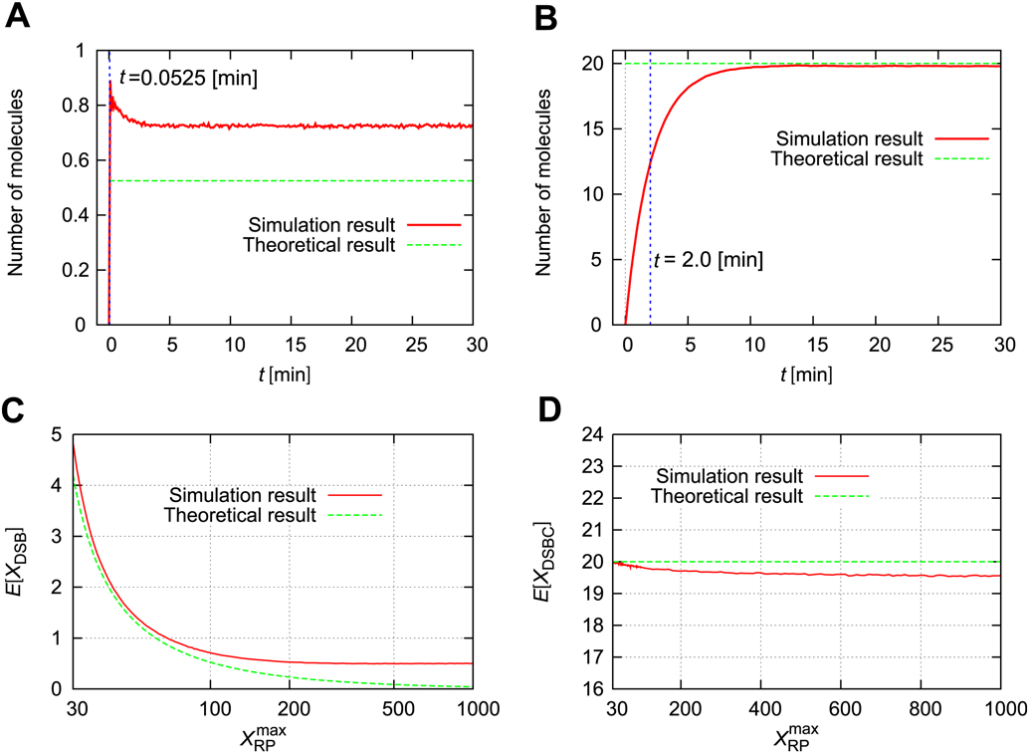
Theoretical mean values of DSBs and DSBCs are suitable for small 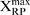. (A) The thick line denotes a simulation result of the ensemble average of the number of DSBs, 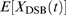. The thin line denotes the theoretical value (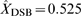). (B) The thick line denotes a simulation result of the ensemble average of the number of DSBCs, 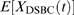. The thin line denotes the theoretical value (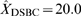). The vertical dotted line denotes the theoretical time 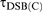. (C, D) Comparisons of theoretical and simulation results for the mean of 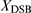 or 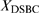 as a function of the maximum number of repair proteins (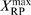) at time *t* = 100 [min]. Initial numbers of molecules are 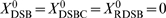, and 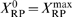.

In this section, we compare simulation and theoretical results of mean values of DSBs and DSBCs. Methods for calculating the theoretical mean values of DSBs and DSBCs are shown in the Materials and Methods section. The precise mechanism of DNA damage processes induced by stress signals has not been clarified yet, and we cannot estimate the stochastic rate constants. In this paper, we assume that the stochastic rate constants 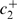, 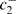, and 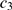 are not affected by stress signals, and their values are defined in Table 1. In addition, we assume the parameter 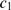 is proportional to the strength of stress signals:
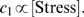
(6)For example, when we define the parameters 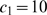, and 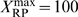, we can estimate the mean numbers of DSBs and DSBCs (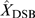 and 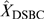). Then the time (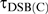) until the 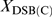 approaches the mean as
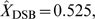
(7)

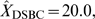
(8)

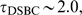
(9)

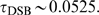
(10)
Figure 3 shows the comparison between simulation and theoretical results. The 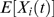 denotes the ensemble average of 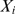 at time *t*. In Figure 3A, we can see that 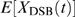 converges on the steady state, but its value is a little different from the theoretical value. The time until the ensemble approaches the steady state (∼2.0 min) is larger than the theoretical value (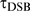). In Figure 3B, 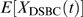 also converges to the steady state, and the value is approximately the same as the theoretical value. However, the time until the ensemble approaches the steady state is approximately 10.0 min. This is larger than the theoretical value (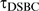). Also, we compare the theoretical and simulation values of the mean numbers of DSBs and DSBCs as a function of the maximum number of repair proteins (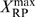) at time *t* = 100 [min]. For a small number of repair proteins (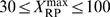), theoretical values can explain the simulation values well. However, as the maximum number of repair proteins increases, the difference between the theoretical and simulation results increases for both 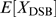 and 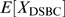.

**Table 1:** Parameters for stochastic models of DSB and ATM modules.

DSB module			
	Description	Units	Values
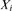	The number of molecules of type *i*	molecules	†
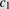	The stochastic rate constant for the DSB production step	molecules min^−1^	10
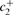	The stochastic rate constant for the DSBC production step	molecules^−1^min^−1^	0.25
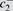	The stochastic rate constant for DSBC failure	min^−1^	0.025
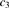	The stochastic rate constant for DSBC success	min^−1^	0.5
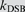	DSB- and DSBC-dependent phosphorylation rate of ATM	molecules^−1^min^−1^	†
τ	The time until the ensemble 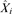 approaches the steady state	min	†
ATM module			
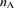	Hill coefficient of ATM*-dependent ATM autophosphorylation rate	–	2
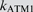	ATM*-dependent autophosphorylation rate of ATM	min^−1^	1.0
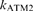	Dephosphorylation rate of ATM*	min^−1^	1.0
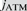	Michaelis constant of ATM*-dependent autophosphorylation rate of ATM	µM	†
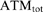	Total concentration of ATM and ATM*	µM	†

† These parameters take multiple values and are defined where appropriate in the text.

### Small differences in the maximum number of repair proteins induce a large difference in DSB generation

**Figure 4:**
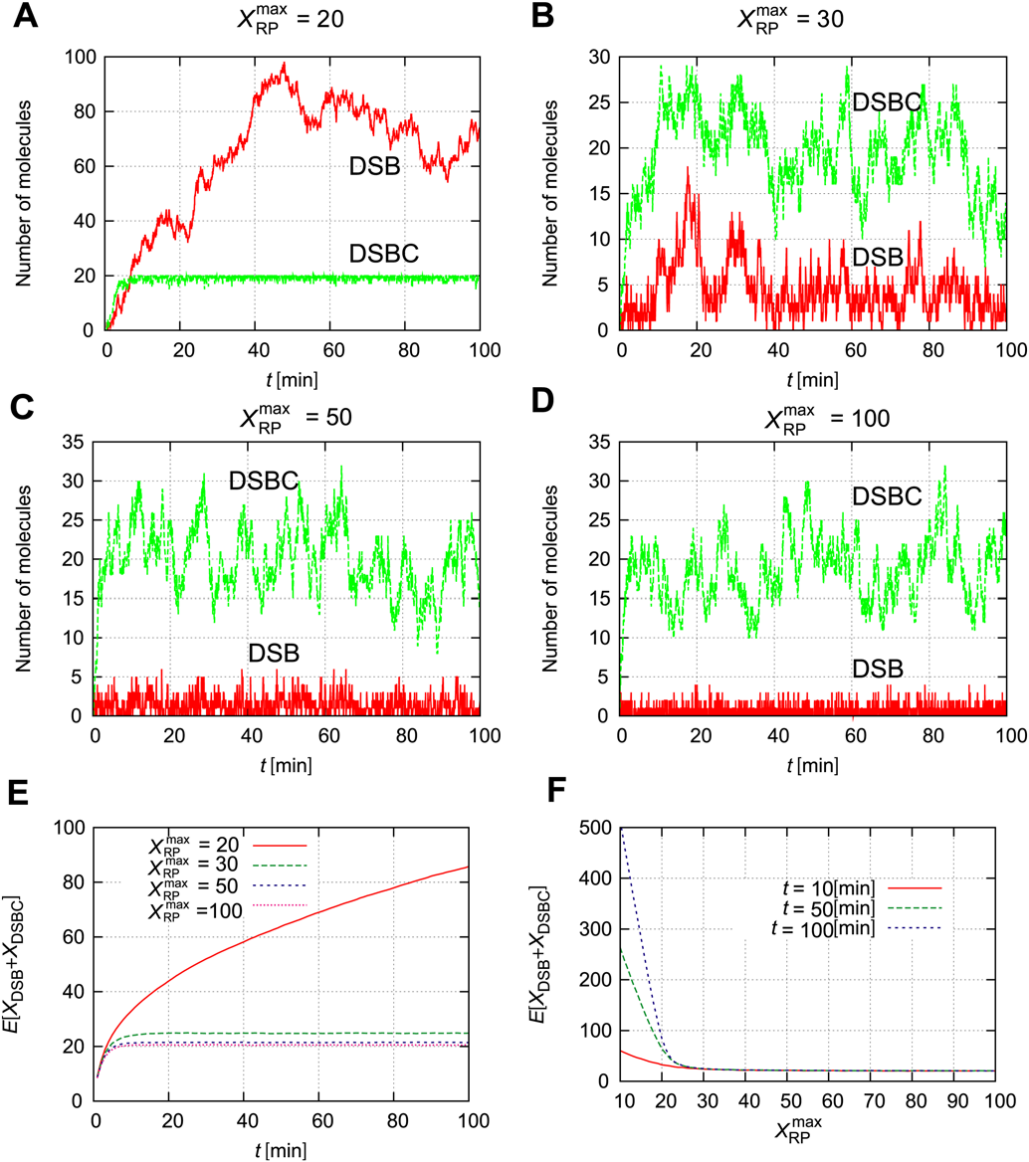
Small differences in 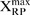 induce a large difference in DSB generation. (A–D) Time courses of the numbers of DSBs and DSBCs with several values for the maximum number of repair proteins 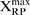. (E) Time-dependent expectation values for several 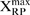. (F) 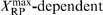 expectation values for several times *t*. Other parameters are as defined in Table 1. Initial numbers of molecules are 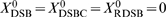, and 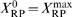.

The time course of the model depends largely on the maximum number of repair proteins, 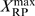. Figure 4 shows the time course of 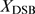 and 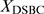 with 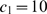. If 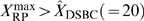, the number of DSBs is very small, and the number of DSBCs approaches 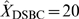. However, the number of DSBCs existing at the same time should be smaller than the maximum number of repair proteins, 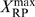. Thus, if the maximum number of repair proteins is smaller than the mean value of 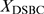, the production rate of DSBs becomes higher than the rate of the DSB-repair processes, and the free repair proteins are given out to repair DSBs; as a result, the number of DSBs increases. Figure 4 shows the time courses of 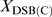 with several maximum numbers of repair proteins. When 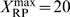, the number of DSBCs is approximately 20, and it cannot exceed 20. Then the number of DSBs gradually increases to nearly 100. On the other hand, when 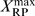 is larger than 30, 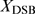 does not continuously increase. As the number of repair proteins 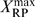 increases, the number of DSBs gradually decreases (Figures 4B, C, and D). Therefore, a time course of the ensemble average of 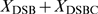 gradually increases for a small number of repair proteins 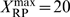, but it converges to a constant value when the number of repair proteins is large (Figures 4E and F). In the following sections, we assume the number of repair proteins is sufficient, and the expected value of 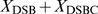 is constant.

### Autophosphorylation of ATM induces bifurcation

**Figure 5:**
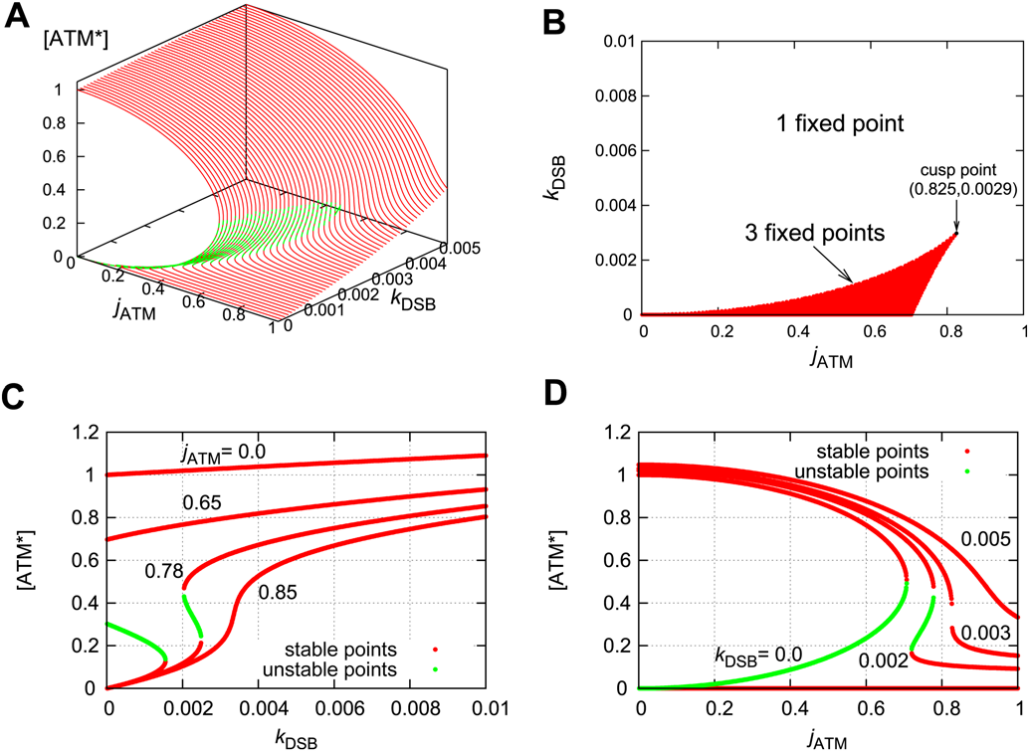
Bifurcation diagrams for the rate constants of 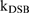 and 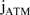. (A) The fixed points as a function of both 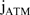 and 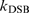. The red lines denote stable steady states, and the green lines denote unstable steady states. (B) The number of fixed points on the parameter space 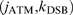. (C) The fixed points of [ATM*] as a function of 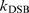 with several 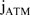. (D) The fixed points of [ATM*] as a function of 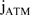 with several 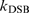. Parameters except for 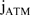 and 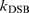 are 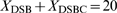, 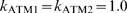, 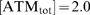, and 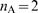.

The ATM module is modeled as follows:
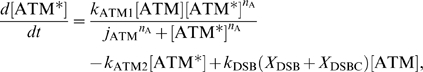
(11)where the total concentration of ATM satisfies 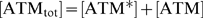, the numbers of DSBs and DSBCs satisfy 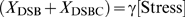, and other rate constants are as described in Table 1. The right-first term denotes autophosphorylation of ATM by ATM* (details are shown in the Materials and Methods section). The true value of the Hill coefficient 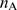 is unknown, but the steady state features of ATM* depend on this value. We assume that this value is 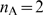 (details of the reasons are described in the Materials and Methods section), which can induce bistability of ATM* as we show in Figure 5. The term 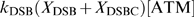 indicates that ATM is phosphorylated by DSBs and DSBCs. Here 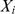 and [ATM] have different dimensions, and therefore 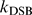 includes a role to adapt the dimensions. If we plot the fixed points above the (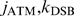) plane, we get the cusp catastrophe surface [21] shown in Figure 5A. This figure shows the steady state concentrations of ATM* and the stability of them. For some parameter regions, there are three fixed points (one is unstable, but the other two are stable). We call these regions bistable regions. We show the parameter regions of three fixed points in Figure 5B. In this figure, we show two bifurcation curves and they meet at a point 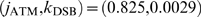, which we call a cusp point. Next, we fix the value of a parameter 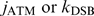, and plot the fixed points as a function of 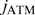 or 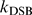. In Figure 5C, we show the fixed points as a function of 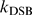 with constant 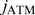 values. As we can estimate from Figure 5B, there exists bistability when the parameter 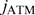 satisfies the condition 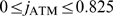. Specifically, we select four values 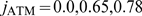, and 0.85. When 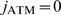, there is a bistable region, but it dominates a very narrow range of 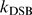. When 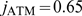, or 0.78, the steady state concentrations of ATM* have bistability. One is irreversible (0.65), and the other is reversible (0.78). For the other value of the parameters, 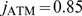, it is monostable as we can expect from Figure 5B. Also, in Figure 5D, we show the fixed points of ATM* as a function of 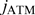 with constant 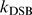 values. As Figure 5B suggests, bistability exists in the condition that 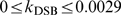. When 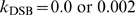, the steady state concentration of ATM* has bistability, but for the other two parameter values it has monostability as we can estimate from Figure 5B. Therefore, the ATM model generates bistability when it contains an autophosphorylation mechanism with the Hill coefficient of 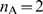 mentioned above.

### The total concentration of ATM determines the bifurcation diagram

**Figure 6:**
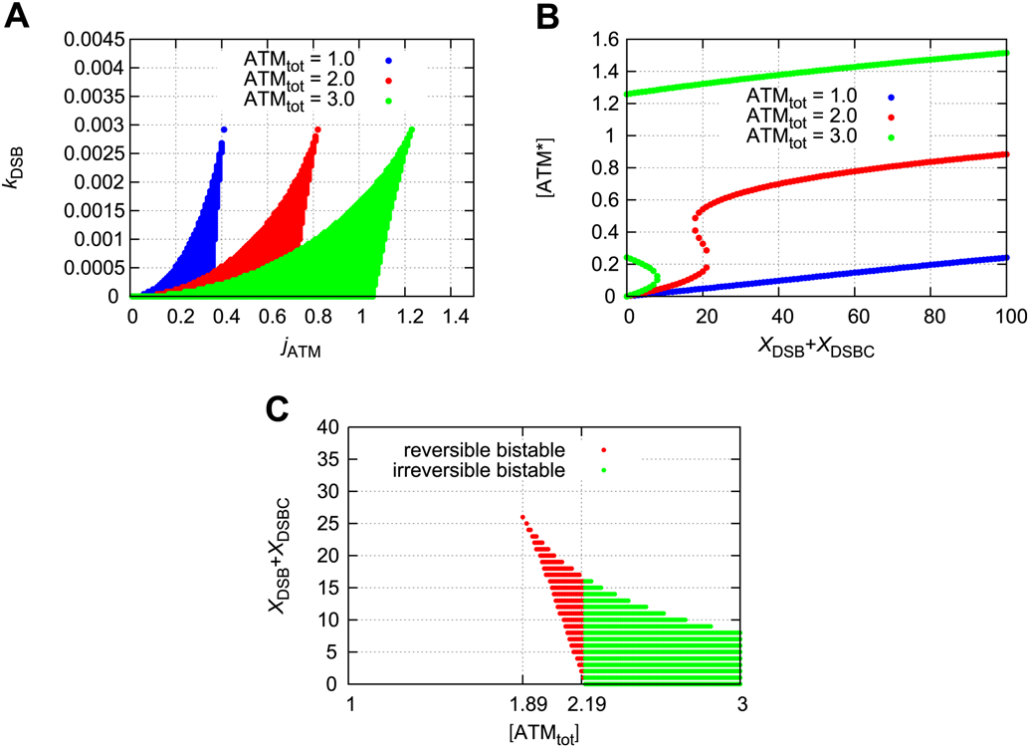
Bistability emerges as the total concentration of ATM increases. (A) Bistable regions for the rate constants 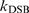 and 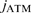 with several total concentrations of ATM. The numbers of DSBs and DSBCs are constant 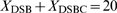. (B) The fixed points of [ATM*] as a function of 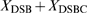 with 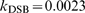 and 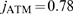. (C) Bistable regions for 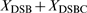 and 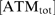. The red points denote reversible bistability and the green points denote irreversible bistability.

Here we consider the effect of the total concentration of ATM on the bifurcation diagram. The bistable regions with the rate constants 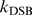 and 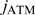 are shown in Figure 6A. When the total concentration of ATM increases, the bistable region expands. For a certain rate constant pair (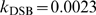 and 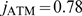), we plot the fixed points as a function of 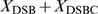 (Figure 6B). The steady state concentration of phosphorylated ATM exhibits irreversible bistability when the total concentration of ATM satisfies 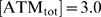 (µM), reversible bistability when 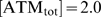 (µM), or monostability when 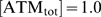 (µM). Therefore, the total concentration of ATM determines the bifurcation diagram. Figure 6C is the bistable region for 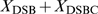 and 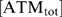. When 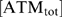 is higher than 1.89 and lower than 2.19, the model exhibits reversible bistability. When 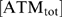 is higher than 2.19, the model exhibits irreversible bistability. In the presence of noise, characteristics of the time-dependent concentration of ATM for the three cases become different. Here the term noise means the repair-process noise for the DSB-production model in equation (1).

### Significance of ATM bifurcation as a DSB sensor

**Figure 7:**
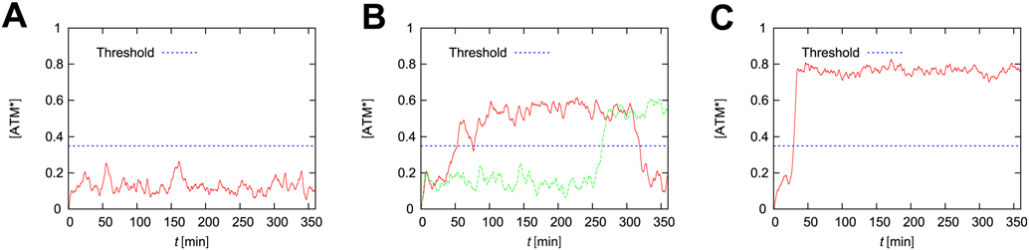
Time courses of the concentration of phosphorylated ATM with the threshold 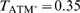. For all figures, the rate constants are 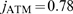 and 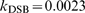. The solid-red lines indicate the time courses. The dashed-blue lines indicate the transition threshold. (A) 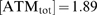 (µM). (B) 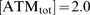 (µM), showing two different simulation results with the same parameters. (C) 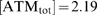 (µM). Initial numbers of molecules are 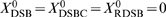, and 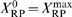. Initial concentrations are 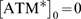 and 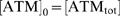.

Here we consider the stochastic case where DSBs are generated stochastically, which means 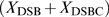 is a stochastic process. To connect these stochastic processes and the deterministic ATM model, we used a hybrid simulation algorithm (see Materials and Methods and reference [22]). We define the parameters of DSBs and DSBCs as in Table 1. In addition, the parameters of the ATM model are 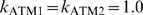. Furthermore, the initial condition of [ATM*] is 0. In this case, the concentration of phosphorylated ATM becomes a stochastic process. Figure 7 shows examples of the time courses of the concentration of phosphorylated ATM with several parameters of 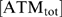.

In the deterministic case, we calculate the steady state [ATM*] for the constant 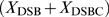, but in the stochastic case 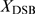 and 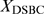 become stochastic processes. Therefore the concentration of [ATM*] fluctuates because of the fluctuations of DSBs and DSBCs. When the total concentration of ATM is small (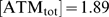 (µM)), the concentration of ATM* is suppressed to a low value. When 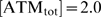 (µM), transitions of the concentration of ATM* between low and high values occur. When 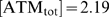 (µM), a transition of the concentration of ATM* from low to high values occurs. These three cases reflect the bifurcation diagrams of deterministic cases. For example, when 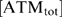 is smaller than 1.89, the fixed points of ATM* are monostable and ATM* fluctuates around a low concentration. However, when 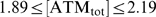, the fixed points of ATM* are reversible and bistable. In this case, once the numbers of DSBs and DSBCs cross over the bifurcation point, ATM* jumps to the higher concentration state. This high concentration state can return to the lower state because of the reversibility of ATM*. When 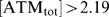, once ATM* jumps to the higher state, ATM* cannot return to the lower state because of the irreversibility of ATM*.

**Figure 8:**
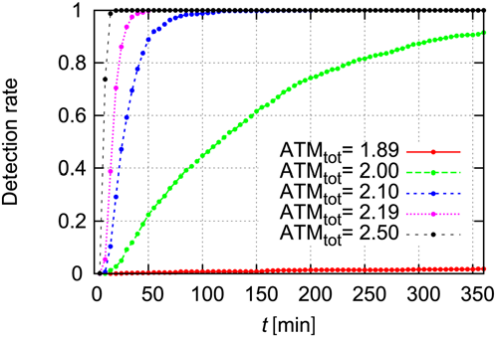
The time to detection of DSBs decreases as the total concentration of ATM increases. The detection rate denotes the fraction of sample paths which pass through the threshold 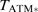 at time *t*. The rate constants are 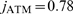 and 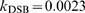. Other parameters are as defined in Table 1. Initial numbers of molecules are 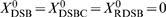, and 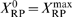. Initial concentrations are 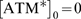 and 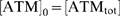.

Here we define the transition threshold 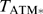 as the value that [ATM*] passes through if transition occurs. When the concentration of ATM* passes the threshold, we assume the DSBs and DSBCs are detected by the ATM module. In Figure 8, we show the detection rate as a function of time. This figure shows that if the total concentration of ATM increases, the detection rate increases, and the response becomes quick. This trend can also be explained from the bifurcation diagrams of the deterministic model. When the total concentration of ATM is between 1.89 and 2.19, ATM* becomes reversible and bistable. As the total concentration increases, the bifurcation point of 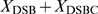 decreases as shown in Figure 6B. Therefore, when the total concentration of ATM is high, ATM* jumps to the higher concentration state even when the numbers of DSBs and DSBCs are small. Thus the detection rate increases.

## Discussion

In this work, we modeled the stochastic repair processes of DSBs and a detection mechanism which is based on the autophosphorylation of ATM. In our first model, we could simulate time-dependent fluctuations of the numbers of DSBs and DSBCs, and we proposed theoretical mean values of DSBs and DSBCs. Depending on the species and strength of stress signals, DSBs may rarely occur. Even in this case, it is suggested that ATM can detect DSBs [16]. We propose that ATM can detect a small number of DSBs by using an ATM autophosphorylation mechanism, which induces bifurcation of ATM*. In the presence of bistability, ATM* exhibited switch-like behavior. Also, we suggested that the total concentration of ATM determines the bifurcation diagrams, and as the total concentration of ATM increases, the detection rate also increases. Therefore, we conclude that the positive auto-regulation works as a sensor of small fluctuating DSBs and amplifier for the detected signals.

### A theoretical method for determining stochastic rate constants

In experiments, dynamics of repair processes of DSBs are still unknown. However, based on the experimental result in which ATM can detect about 18 DSBs in a cell, we defined the parameter values of 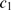, 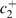, 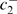, and 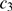 which induces approximately 20 DSBs plus DSBCs. Then we considered how ATM detects these small numbers of DSBs and DSBCs. To define these four parameters, we described theoretical mean values of DSBs and DSBCs by using the four parameters (equations (19) and (20) in Materials and Methods). Interestingly, the mean number of DSBCs (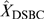) only depends on the production rate of DSBs (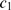) and the success rate of DSBCs (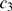). On the other hand, the mean number of DSBs (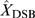) complexly depends on all rates and the maximum number of repair proteins (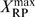) which is inversely proportional to 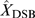. We compared these theoretical results and simulation results. As expected, 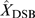 gradually decreases as 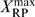 increases (Figure 3). However, the difference between theoretical and simulation results for 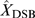 and 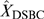 increases as 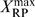 increases. More specifically, the theoretical 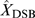 converged to 0, but the simulation result converged to 0.5. This may be because of the fact that in the simulation results 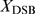 and 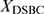 are small and they are stochastic processes depending on hazard functions 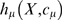, which define the reaction rates. Therefore, mean-field approximation of theoretical results cannot evaluate the true mean numbers of DSBs and DSBCs. For example, 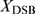 usually becomes 0 or 1 when 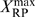 is large. In this situation, if 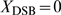, the hazard function 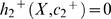, and repair processes are independent of the repair proteins. Therefore, DSBs can be generated regardless of the number of repair proteins. If 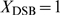, the hazard function 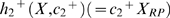 becomes very large, and a generated DSB is immediately repaired. In addition, when 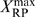 is very large, each process occurs at approximately the same rate. Thus the mean value of DSBs does not converge to 0 but some other value between 0 and 1.

Many experiments should be done to confirm our results. We have to observe the numbers of DSBs and DSBCs as functions of time in a single cell. The number of repair proteins characterizes repair processes of DSBs and also should be observed. Even when these observations are successful, it is difficult to estimate the stochastic rate constants for repair processes. For example, when we get the time until the number of DSBCs approaches the mean 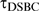, we can estimate the stochastic rate constant 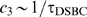 (see Materials and Methods). Then we can also estimate the stochastic rate constant 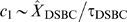. The other stochastic rate constants 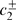 cannot easily be estimated by using 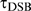 and 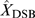. However, if we assume that 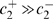, we can estimate the stochastic rate constants as
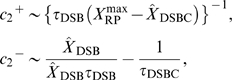
where the maximum number of repair proteins should be 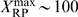 because of the discrepancy between the theoretical and simulation results. Details are further discussed in the Materials and Methods section.

### Biological interpretations for the emergence and consequences of bistability of ATM*

Here we discuss biological interpretations of our results. First, we consider the significance of the ATM autophosphorylation mechanism. The autophosphorylation of ATM increases the active ATM proteins, which again increases the active ATM proteins. Thus it works as a positive feedback loop of ATM*. This mechanism is described by a nonlinear Hill equation as we show in the Materials and Methods section. In this equation, a Hill coefficient 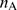 is an important factor for nonlinearity. For example, as the Hill coefficient 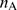 increases, the nonlinearity of the equation also increases. For our model, we assume that inactive ATM proteins exist as dimers and two ATM* proteins autophosphorylate the dimer which is indicated by Bakkenist et al. [16]. In this case, we can generally define the Hill coefficient as 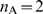, which means that two ATM* proteins bind to an ATM dimer protein and phosphorylate it. Then bistability emerges in this system for appropriate values of parameters. In Figure 5, we consider conditions for parameters 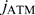 and 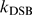 where bistability arises. Thus, our results indicate an physical importance of the ATM dimer proteins, in that the dimer leads to the high value of the Hill coefficient which emerges bistability of the steady state concentration of ATM*.

Biological importance of a positive feedback loop is considered by Xiong et al. [4]. They provide experimental evidence that in a physiological process of cell fate induction, *Xenopus* oocyte maturation, a bistable signalling system of a MAPK cascade converts a transient stimulus into a reliable, self-sustaining, effectively irreversible pattern of protein activities. Also, in DNA damage response processes, our model suggests that bistability, which is generated from autophosphorylation, amplifies transient damage signals (DSBs and DSBCs), and sustains the active state in the presence of DSB and DSBC noise. Other experiments indicate that bistability arises when there is a positive feedback loop in gene regulation networks [5], [23], [24]. In general, it is suggested that hysteresis, which is caused by bistability, leads to robustness against noise rather than an ultra-sensitive response in which the system is monostable [25]. Therefore, biological meanings of bistability which have two states (on and off states) will be (i) amplifying the input signal by the mechanism of hysteresis, and (ii) maintaining an on or off state in the presence of noise.

Other experimental results suggest that positive feedback of ATM contributes to genomic stability [26]. In the experiments, authors indicate that the initial DNA damage signal induces ATM activation and recruitment, and results in early H2AX phosphorylation immediately adjacent to DSBs. Phosphorylated H2AX then binds to MDC1 (mediator of DNA damage checkpoint protein 1) and causes additional activation of ATM. ATM then phosphorylates its substrates, resulting in checkpoint activation and DNA repair [26]. In the experiments, authors concluded that positive feedback of ATM amplifies the damage signal and it is vital in controlling proper DNA damage response and maintaining genomic stability [26]. Assumptions of our model are different from those of the above experiments in that positive feedback is caused by the autophosphorylation of ATM. However, our model also suggested that positive feedback of ATM amplifies DSB and DSBC damage signals and maintains a state in the presence of fluctuation of DSBs and DSBCs. Therefore, our model provides theoretical bases of the genomic stability which is indicated in the previous experimental results.

### Effects of a negative feedback for the phosphorylated ATM concentration

**Figure 9:**
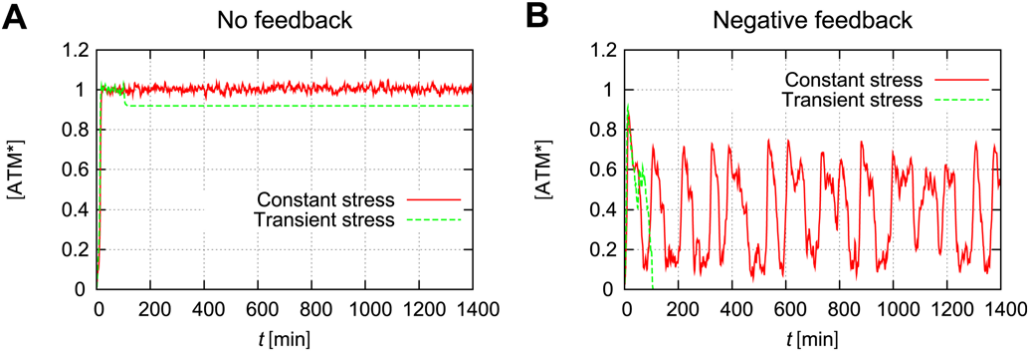
Negative feedback from p53 induces oscillations of ATM*. Models are defined in the Materials and Methods section (equations (37)–(38)). (A) Time courses of ATM* without feedback from p53. (B) Time courses of ATM* with negative feedback from p53. For both cases, the total concentration of ATM is 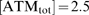, which induces irreversible bistability. Constant stress means that 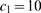 indefinitely. Transient stress means that 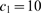 for 0≤*t*≤100 [min], but 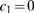 for *t*>100 [min]. The rate constants are 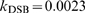, 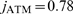, and 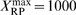. Initial numbers of molecules are 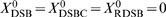, and 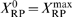. Other parameters and initial concentrations are defined in Table 1 and in the Materials and Methods section.

Recent studies suggest that the p53/Mdm2 negative feedback loop generates oscillation of p53 and Mdm2 [27], [28]. These pulses are initiated by DNA damage and the signaling kinase, ATM. Batchelor et al. suggest that the negative feedback between p53 and ATM, via Wip1, is essential for maintaining the uniform shape of p53 pulses [29]. We consider how this negative feedback affects ATM's dynamical behaviour. For simplicity, we only consider the case that the fixed points of ATM* have irreversible bistability in which the detection time is short. In the absence of negative feedback from p53, once ATM* is activated to the higher state, the concentration of ATM* is sustained with the same value because of the irreversibility. This phenomenon means that ATM works as a memory module of DNA damage. Figure 9A shows time courses of the concentration of ATM* without negative feedback. In this case, even when we add some transient stress (0≤*t*≤100 [min]), the concentration of ATM* sustains high values. Therefore, once the DNA damage is detected, the concentration of ATM* maintains high values without further DNA damage. In the presence of negative feedback from p53, the concentration of ATM* exhibits instability, and it oscillates under the constant stress. Figure 9B shows time courses of ATM* with a negative feedback loop. The concentration of ATM* is oscillating under some constant stress. However, it suddenly decreases after some transient stress (0≤*t*≤100 [min]). Detailed models are described in the Materials and Methods section. These results indicate that when DNA damage occurs, ATM detects DSBs as quickly as possible, and the concentration of ATM* becomes high. This high concentration state sustains until p53 is activated to suppress ATM phosphorylation. In addition, the concentration of p53 oscillates, which results from reactivation from ATM when DNA damage persistently exists.

### Further challenges for the initial DNA damage response model

Experiments are needed to confirm whether ATM* has bistability which is caused by the autophosphorylation of ATM. As we show above, bistability collapses when the negative feedback from p53* exists. Therefore the phosphorylation of p53 should be blocked when we identify bistability. In addition, there are many signaling pathways from ATM [10], [30]. The phosphorylation sites of p53 vary depending on the DNA damage agents, and it has not been known whether p53 is always phosphorylated and generates negative feedback to ATM for all stress signals. Quantitative data of the concentrations of p53 and ATM under several stress signals have not been observed enough. Further observations might provide new insight into DNA damage responses and signaling processes after damage. In addition, other experiments suggest that after exposure to H_2_O_2_, the p53 and ERK proteins are phosphorylated, which induce apoptosis or survival, respectively [31]. Also, the decision of the two exclusive fates is stochastically determined in independent cells. In this paper, our model only addresses the initial responses to DNA damage, but we may expand this model and clarify the mechanism of stochastic and exclusive decision of apoptosis.

## Materials and Methods

### Theoretical description of the mean numbers of DSBs and DSBCs

We can estimate the production rates of molecules per unit time. Here we denote such rates as 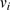, where *i* is a molecular type.
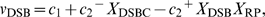
(12)

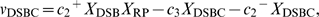
(13)

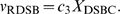
(14)The number of molecules for 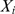 is time dependent (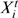), but some of them are assumed to be in the mean convergence, 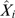, meaning
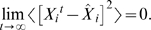
(15)


If there are enough repair proteins, we can assume that the numbers of DSBs, DSBCs, and free RP are in the mean convergence, and we describe them as 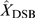, 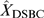, and 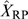. In addition, when we assume that the maximum number of repair proteins (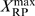) is fixed and larger than 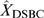, we can estimate 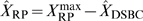. In this situation, the mean production rates 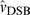 and 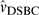 approach their steady states and become 0, and the mean rate 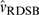 approaches 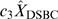. Here we estimate mean values of DSBs and DSBCs. In the steady states, 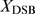 and 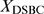 satisfy
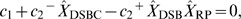
(16)

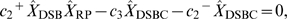
(17)

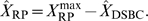
(18)


When we add equation (16) to equation (17), we have the mean number of DSBCs by using the stochastic rate constants:
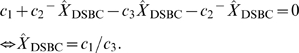
(19)


Then we substitute equations (18) and (19) to (16), and we can describe the mean number of molecules for DSBs by using the stochastic rate constants and the maximum number of repair proteins 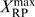:
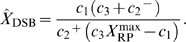
(20)


Because the number of DSBs which are generated per unit time is 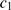, it takes at least 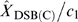 to generate enough DSB(C)s. Thus the time until the number of DSBCs approaches the mean, 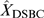, is calculated by
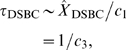
(21)and that for DSBs is calculated by
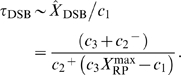
(22)


### Steady state analysis

Here we show the calculation method of steady states and their stability [21]. The steady state concentration of ATM* is calculated by equation (11). Here we denote it as:
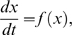
(23)where *x* denotes the concentration of ATM*. The steady states of *x* satisfy *dx*/*dt* = 0 and they are solutions of
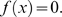
(24)


When we set a solution of *f*(*x*) = 0 as 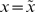, the stable steady states satisfy
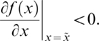
(25)


We used Mathematica (version 5.2) to solve equation (24) and estimate the stability of solutions. The source code to find steady state concentrations of ATM* and the stability of them is included in Text S1.

### The Gillespie algorithm

In this section, we introduce a method for calculating stochastic repair processes of DSBs. The Gillespie algorithm is one of the famous simulation methods [18], [20]. It is clear that the time course of the state of the reaction system (the number of molecules of each type) can be regarded as a continuous Markov process with a discrete state space, because of the fact that the reaction hazards depend only on the current state of the system. Here we show a method for stochastic simulation of the time-evolution of the system.

In a reaction system which contains *m* reactions, the hazard of reaction 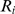 obeys 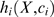, so the total hazard for all reactions is
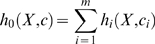
(26)


In this reaction system, each chemical reaction occurs following a Poisson process, and therefore time to the next reaction (δτ) obeys an exponential distribution with parameter 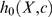, thus δτ obeys
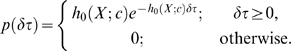
(27)


Also, the probability that the *i*th reaction occurs after this time interval δτ is proportional to 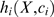, independent of the time to the next event. Therefore, the reaction type will be *i* with probability 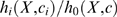. Using the time to the next reaction and the reaction type, the state of the system can be updated, and simulation can continue. This simulation procedure was first proposed by Gillespie and is known as the “Gillespie algorithm” (or “Gillespie's direct method”) [20]. The concrete procedure of this algorithm is as follows:

### Gillespie's direct method

1. Set the rate constants 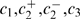 and initial numbers of molecules 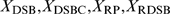 at time *t* = 0.
2. For each reaction, calculate 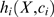 (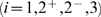) based on the current state, 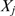 (*j* = DSB, DSBC, RP, or RDSB).
3. Calculate a combined reaction hazard 
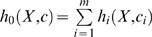
.
4. Calculate the time to the next event δτ which follows the exponential distribution.
5. Set *t* = *t*+δτ.
6. Select a reaction type, *i*, based on probabilities 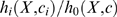 for each reaction *i*.
7. Update *X* according to reaction *i*.
8. Output *X* and *t*.
9. If 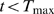, return to step 2.

The source code of this algorithm is included in Text S1.

### Hybrid simulation algorithm

The stochastic behavior of DSBs can be calculated by the Gillespie algorithm [19], [20]. In equation (11), the numbers of DSBs and DSBCs, 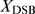 and 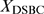, are random variables. Here, we have to connect the continuous equations and the stochastic ones to solve the ATM sensor equation (equation (11)). In other words, the time until the next reaction occurs in the discrete (stochastic) regime, δτ, is not constant, and it does not match the time step of the numerical algorithm in the continuous regime, δ*t*. To settle the problem, we used a hybrid simulation method [18], [22] in which some processes are simulated discretely while other processes are handled in a continuous manner by differential equations. Kiehl et al.'s method treats the effects of round trip conversions between discrete and continuous variables [22]. In our model, the numbers of DSBs and DSBCs affect the phosphorylation of ATM, but ATM proteins do not affect repair processes of DSBs, and thus the discrete reactions do not depend on the continuous reactions. That is to say, there are no time-varying hazard functions which are affected by ATM. Thus the hybrid simulation algorithm for our model can be simplified to the following procedure. In this algorithm, the time step of the numerical algorithm in the continuous regime is fixed as δ*t*. At first, we determine initial conditions of both discrete and continuous values, and calculate reaction hazards of discrete reactions 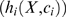 at time *t*. From these hazards, we can calculate the time to the next reaction (δτ), and the reaction type *i* of the discrete regime by using Gillespie's direct method [20]. After these preparations, we compare the step sizes of discrete and continuous regimes, and select a smaller step size to update the simulation time as *t*: = *t*+min{δτ,δ*t*}. For both cases, we update the continuous variables to the values for the new time by using a numerical solution algorithm for ordinary differential equations (Euler, Runge-Kutta, etc.). Only when some discrete reaction has occurred, we update the discrete variable according to the reaction type *i*. The concrete procedure of this algorithm is as follows:

1. Set time *t* = 0, and determine initial conditions of 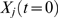 (*j* = DSB, DSBC, RP, RDSB) and [ATM*](*t* = 0).
2. Calculate the discrete reaction hazards 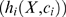 at time *t*.
3. From the hazards of the discrete reactions, select a discrete time step size δτ and type of reaction *i* by using Gillespie's direct method.
4. If δτ>δ*t* (no discrete reaction has taken place), set *t* = *t*+δ*t*, and update the continuous variables to the values appropriate for this new time.
5. If δτ≤δ*t* (some discrete reaction has occurred), set *t* = *t*+δτ, update the continuous values to those appropriate for this new time, and update the discrete variables according to the reaction type which is selected in step 3.
6. If *t* is less than the simulation duration, return to step 2.

The source code of this algorithm is included in Text S1.

### Hill equation for the autophosphorylation mechanism of ATM

In this section, we introduce a detailed description of the autophosphorylation mechanism of ATM in equation (11). Thus we only focus on the first term of the equation, and clarify the meanings of the Hill coefficient 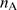 and the parameters 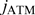 and 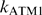. It is suggested that when ATM is phosphorylated by DNA damage, the activated ATM phosphorylates the inactive ATM [16], [28]. The inactive ATM exists as dimers which consist of two ATM* [16]. We assume that 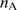 molecules of the active ATM (ATM*) bind to the ATM dimer (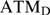), becomes a complex 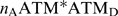, and the dimer is phosphorylated to the active form two ATM* molecules. This scheme is described as follows:
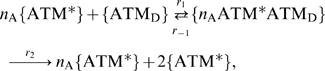
where {} denotes molecular species, 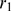 denotes the binding rate of ATM* to 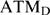 (→), 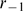 denotes the disassociation rate (←), and 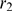 denotes the phosphorylation rate from the binding form. The conservation equation satisfies
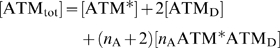
(28)where the relationship between the concentrations of ATM and 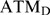 is
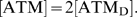
(29)


The reaction rate law for the complex 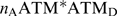 is
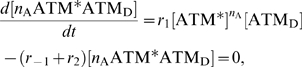
(30)where we assume the quasi-steady state approximation, and the right term of this equation becomes zero. Equations (28) and (30) allow us to calculate the concentration of the complex as
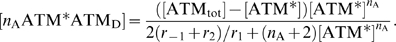
(31)


Thus the rate law of the concentration of ATM* (only for autophosphorylation mechanism) is
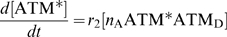
(32)

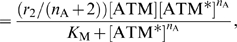
(33)where we assume 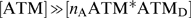 and 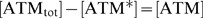. In addition, 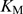 denotes
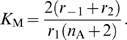
(34)


When we compare equations (11) and (34), the rate constants satisfy
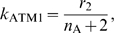
(35)

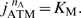
(36)


In our model, we do not define the parameters, 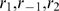, but directly define the parameters 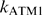 and 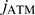 as we show in the next section.

### Parameter values

Parameter values which are used in our model are shown in Table 1. The true units of DSBs and DSBCs are not “molecules” but “sites”. However, we can deal with them as molecules in the DSB-repair process model, and therefore we simply denote their units as “molecules”. In previous experiments, it is suggested that ATM is autophosphorylated in 15 min after exposure to 0.5 Gy IR, which causes only 18 DNA double-strand breaks in a cell [16]. In addition, equation (20) indicates that if the maximum number of repair proteins is large, the mean number of DSBs becomes small. This result is also supported by our simulation results in Figure 4D. Therefore, we estimate that the main products in DNA damage processes are DSBCs which phosphorylate ATM. Based on this assumption, we estimate the theoretical time until DSBCs reach the steady state 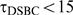 (min) because the time until ATM is autophosphorylated is within 15 min. Simulation results indicate that when 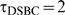 (min), the mean number of DSBCs becomes the steady state in 15 min (Figure 3B). In this case, the stochastic rate constant 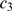 becomes 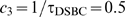 (see equation (21)). Also we estimate the mean number of DSBCs as 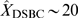, and define the stochastic rate constants as 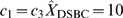 (see equation (19)). When the maximum number of repair proteins is large, both 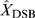 and 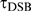 become small, and the stochastic rate constants 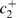 and 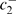 have small effects on 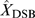 and 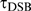. Here we assume that the association rate of repair proteins (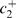) is larger than the dissociation rate (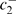), as the previous work suggested [28]. Thus we simply define these parameters as 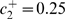 and 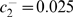.

As we showed in the previous section, the Hill coefficient 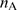 denotes the number of molecules which bind to inactive ATM. Experimental results indicate that inactive ATM exists as a dimer [16], and thus we can assume two phosphorylated ATMs bind to an inactive ATM dimer. Therefore we estimate the Hill coefficient as 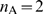 in our model. In addition, the rate constants 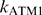 and 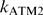 are estimated to be 0.1∼10 in Ma et al.'s work [28]. Their model successfully explained the previous experimental results [28], [32]. Thus we simply selected two values 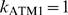 and 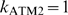 which are between 0.1 and 10.

After the above preparations, we finally estimated parameters 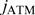, 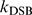, and 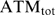. To begin with, we fixed the total concentration of ATM as 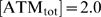 (µM), and calculated the steady state concentrations of ATM* as functions of 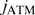 and 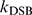 (Figure 5). Then we found the region of 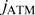 and 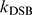 where bistability of ATM* exists. We selected the rate constants as 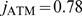 and 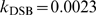 from bistable regions, because we predict that bistability plays an important role in DNA damage detection. Next, we studied dependencies of the total concentration of ATM on bistability (Figure 6). This figure suggests that if bistability of ATM* exists, the total concentration of ATM needs to be larger than 1.89 µM.

### ATM/p53 negative feedback model

A negative feedback model of ATM is defined as follows:
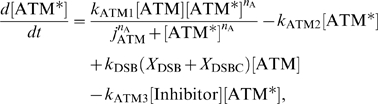
(37)

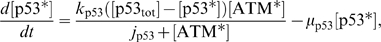
(38)

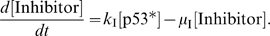
(39)


In the ATM equation, 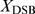 and 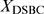 are stochastic processes which are calculated from scheme (1). We assume that the ATM proteins are directly dephosphorylated by the inhibitor proteins (the dephosphorylation rate is 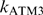). The p53 proteins are phosphorylated by the activated ATM proteins (the phosphorylation rate of p53 is 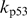 and the Michaelis constant of it is 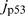). Then the inhibitor proteins are induced by activated p53 (the induction rate of the inhibitor proteins is 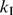). We do not consider the gene expression of p53 for simplicity. Parameters used in Figure 9 are as follows: 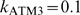, 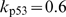, 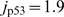, 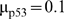, 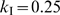, 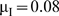, and 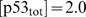. These parameters are selected such that the system triggers p53 pulses (leading to the ATM* pulses) which is indicated in the previous experiments [29]. The initial concentrations are 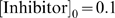 (µM), 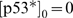 (µM), and 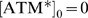 (µM). Other parameters are defined in Table 1 or in the main text.

### Estimation of the stochastic rate constants

Here we show how we can estimate the stochastic rate constants from experimental results of (i) the mean numbers of DSBs and DSBCs and (ii) the time until the numbers of DSBs and DSBCs approach their mean. Theoretical values of (i) and (ii) are as follows:
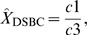
(40)

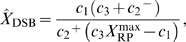
(41)

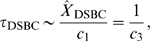
(42)

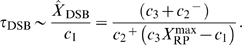
(43)


Examining the right hand terms of the above equations, 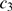 is easily estimated by equation (42):
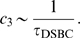
(44)


Therefore, 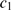 can be estimated by equation (40):
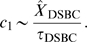
(45)


We can directly estimate 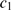 and 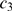 from equations (40) and (42), but 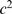 cannot be estimated directly from equations (41) and (43) because of the complexity of those equations. However if we assume that 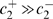, the approximate values of 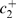 can be estimated as follows:
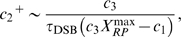
(46)where we use 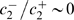 when 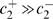. When we substitute 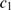 and 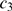 of equations (44) and (45) into equation (46), we get
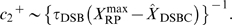
(47)


In addition, when we substitute equations (44), (45), and (47) into equation (41), 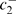 can be approximated as
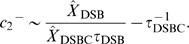
(48)


In equation (47), we have to estimate a value of 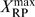. However, the discrepancy between theoretical and simulation results makes it difficult to estimate the value. In particular, even if there are enough repair proteins, the mean number of DSBs from a simulation converges to 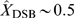, which is not 0 as theoretical results predict. In equation (41), 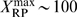 when the mean number of DSBs satisfies 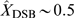.

## Supporting Information

Text S1 This file includes 4 source codes of C program and 1 source code of Mathematica to calculate our models, which use Gillespie algorithm or hybrid simulation algorithm (for C codes), and steady state analysis (for a Mathematica code). Details are shown in the README text.
(0.01 MB ZIP)

## References

[1] Alon U (2006). An introduction to systems biology. Design principles of biological circuits.

[2] Ferrell JE, Machleder EM (1998). The biochemical basis of an all-or-none cell fate switch in *Xenopus Oocytes*.. Science.

[3] Angeli D, Ferrell JE, Sontag ED (2004). Detection of multistability, bifurcations, and hysteresis in a large class of biological positive-feedback systems.. Proc Natl Acad Sci USA.

[4] Xiong W, Ferrell JE (2003). A positive-feedback-based bistable ‘memory module’ that governs a cell fate decision.. Nature.

[5] Gardner TS, Cantor CR, Collins JJ (2000). Construction of a genetic toggle switch in *Escherichia coli*.. Nature.

[6] Elowitz MB, Levine AL, Siggia ED, Swain PS (2002). Stochastic gene expression in a single cell.. Science.

[7] Arkin A, Ross J, McAdams HH (1998). Stochastic kinetic analysis of developmental pathway bifurcation in phage λ-infected *Escherichia coli* cells.. Genetics.

[8] Ozbudak EM, Thattai M, Lim HN, Shraiman BI, van Oudenaarden A (2004). Multistability in the lactose utilization network of Escherichia coli.. Nature.

[9] Choi PJ, Cai L, Frieda K, Xie XS (2008). A stochastic single-molecule event triggers phenotype switching of a bacterial cell.. Science.

[10] Kastan MB, Lim DS (2000). The many substrates and functions of ATM.. Nat Rev Mol Cell Biol.

[11] Boveris A, Chance B (1973). The mitochondrial generation of hydrogen peroxide.. Biochem J.

[12] Mello Filho AC, Meneghini R (1984). In vivo formation of single-strand breaks in DNA by hydrogen peroxide is mediated by the Haber-Weiss reaction.. Biochem Biophys Acta.

[13] Samuni AM, DeGraff W, Krishna MC, Mitchell JB (2001). Cellular sites of H_2_O_2_-induced damage and their protection by nitroxides.. Biochim Biophys Acta.

[14] Cantoni O, Giacomoni P (1997). The role of DNA damage in the cytotoxic response to hydrogen peroxide/histidine.. Gen Pharmac.

[15] Lee JH, Paull TT (2004). Direct activation of the ATM protein kinase by the Mre11/Rad50/Nbs1 complex.. Science.

[16] Bakkenist CJ, Kastan MB (2003). DNA damage activates ATM through intermolecular autophosphorylation and dimer dissociation.. Nature.

[17] You Z, Bailis JM, Johnson SA, Dilworth SM, Hunter T (2007). Rapid activation of ATM on DNA flanking double-strand breaks.. Nat Cell Biol.

[18] Wilkinson DJ (2006). Stochastic Modelling for Systems Biology. Mathematical and Computational Biology Series.

[19] Gillespie DT (1976). A general method for numerically simulating the stochastic time evolution of coupled chemical reactions.. J Comput Phys.

[20] Gillespie DT (1977). Exact stochastic simulation of coupled chemical reactions.. J Phys Chem.

[21] Strogatz SH (1994). Nonlinear dynamics and chaos. With applications to physics, biology, chemistry, and engineering.

[22] Kiehl TR, Mattheyses RM, Simmons MK (2004). Hybrid simulation of cellular behavior.. Bioinformatics.

[23] Becskei A, Séraphin B, Serrano L (2001). Positive feedback in eukaryotic gene networks: cell differentiation by graded to binary response conversion.. EMBO J.

[24] Isaacs FJ, Hasty J, Cantor CR, Collins JJ (2003). Prediction and measurement of an autoregulatory genetic module.. Proc Natl Acad Sci USA.

[25] Rao CV, Wolf DM, Arkin AP (2002). Control, exploitation and tolerance of intracellular noise.. Nature.

[26] Lou Z, Miner-Dykhouse K, Franco S, Gostissa M, Rivera MA (2006). MDC1 maintains genomic stability by participating in the amplification of ATM-dependent DNA damage signals.. Molecular Cell.

[27] Lev Bar-Or R, Maya R, Segel LA, Alon U, Levine AJ (2000). Generation of oscillations by the p53-Mdm2 feedback loop: a theoretical and experimental study.. Proc Natl Acad Sci USA.

[28] Ma L, Wagner J, Rice JJ, Hu W, Levine AJ (2005). A plausible model for the digital response of p53 to DNA damage.. Proc Natl Acad Sci USA.

[29] Batchelor E, Mock CS, Bhan I, Loewer A, Lahav G (2008). Recurrent initiation: a mechanism for triggering p53 pulses in response to DNA damage.. Molecular Cell.

[30] Kurz EY, Lees-Miller SP (2004). DNA damage-induced activation of ATM and ATM-dependent signaling pathways.. DNA Repair.

[31] Nair VD, Yuen T, Olanow CW, Sealfon SC (2004). Early single cell bifurcation of pro- and antiapoptotic states during oxidative stress.. J Biol Chem.

[32] Lahav G, Rosenfeld N, Sigal A, Geva-Zatorsky N, Levine AJ (2004). Dynamics of the p53-Mdm2 feedback loop in individual cells.. Nat Genet.

